# Sustainability assessment of biogas production from buffalo grass and dung: biogas purification and bio-fertilizer

**DOI:** 10.1007/s13205-018-1170-x

**Published:** 2018-02-26

**Authors:** Ajcharapa Chuanchai, Rameshprabu Ramaraj

**Affiliations:** 1 School of Renewable Energy, Maejo University, Chiang Mai, 50290 Thailand; 2 Energy Research Center, Maejo University, Chiang Mai, 50290 Thailand

**Keywords:** Buffalo grass, Buffalo dung, Biogas production, Methane enhancement

## Abstract

Biomass from wetland aquatic grass and buffalo grass can be exploited for biogas production, because this substrate is plenteous and does not compete with food production. In this study, the grass substrate was physically pretreated by boiling with different retention time to increase its biodegradability and was examined in batch mode. Boiling pretreatment suggested that 100 °C with 2 h retention time was the best condition. The results showed that the optimum grass concentration in the 1:1 ratio of co-digestion mixture with manure produced the highest methane yield. The results suggested that co-digestion of buffalo grass and buffalo dung was a promising approach for improving biogas production. This study was achieved the upgraded biogas through biological purification contained 90.42% CH_4_ 8.04% CO_2_ 1.43% O_2_ and 0.11% other trace gases—a remarkable performance based on an efficiency criteria. Furthermore, the digestate has high nutrient concentrations that can potentially use as fertilizer.

## Introduction

The environmental and global warming consciousness has become an important policy in all countries around the world. Furthermore, the fossil fuel use has been related to some alarming environmental problems such as global warming and climate change (Tsai et al. 2016; Vu et al. 2017). These increasing demands for energy, together with the weakening and limited source of fossil fuels, together with the harmful impacts in the environment, are the reasons industries and governments worldwide are pursuing renewable alternatives. Bioenergy, a renewable energy sources, draws responsiveness due to its accessibility and low carbon dioxide emission (Ramaraj et al. 2016a, b ,c). Thai government has increasingly given an importance on how to solve this problem issues among the first priority in local development.

At present, many agencies have focused on renewable energy such as solar energy, wind energy, hydroenergy and geothermal energy. Renewable sources of energy and consumer products are required for sustainable development of modern society (Unpaprom et al. 2017; Vu et al. 2017). Energy demand required to meet the economic growth of Thailand is growing higher in every year (Dussadee et al. 2017). Accordingly, Thailand has a huge potential to develop renewable energy from biomass as the country has an abundant agriculture sources such as raw materials from crops and livestock that can be used to produce biogas, specifically methane gas, through the decomposition of organic matter in the system (Dussadee et al. 2014; Vu et al. 2018).

Plant biomass is the main source of renewable materials on Earth and represents a potential source of renewable energy and bio-based products (Guo et al. 2015; Wannapokin et al. 2017). Animal manures have been used as a resource of excellent material for anaerobic digestion (AD) with clear environmental benefit, especially for buffalo dung. Since Thailand economy depends mainly on agriculture activities, therefore, utilization of natural resources for energy production is an extremely important issue. Agricultural residues from the agricultural sector, agriculture industry and grassland biomass are usually used as feed materials in anaerobic digestion systems in Thailand which are suitable in numerous ways for producing energy. There are so many types of grasses that are popularly grown in Thailand (Dussadee et al. 2017). Deb et al. (2016) stated that the buffalo grass, traditionally raised in a mixed crop livestock system, has played an important role over the centuries, especially in Asia, for the lives of millions of people, by ensuring work power and food at the end of their career as work animals.

Buffalo grass a tropical and invasive growing plant in rural area has only value to be feedstock for animal feeding. These exotic grass weeds are overgrown in abundantly available resources in the Northern region of Thailand. It needs to cut down and removed frequently for fire hazard, and disease and vector controls (Sahoo et al. 2017). The present study investigates the possibility of buffalo grass as a feedstock for biogas production using certain pretreatment. Rösch et al. (2013) stated that grass is converted to silage that can be used as feedstock for anaerobic digestion. This can be utilized as raw materials for an environmentally friendly renewable energy, more specifically for biogas production. Additionally, the use of grassland biomass for the biogas production is currently the common practice. Biogas application includes ensuring energy security, decreasing carbon emission and improving economic activity. It can be produced by a single raw material such as pig manure, cow manure and buffalo manure. Furthermore, Thailand is being in top 11 in the countries of Asia for buffalo population.

In present, the production of biogas has been evolving to enhance the efficiency like co-digestion of buffalo dung with grass. Co-digestion of buffalo grass (para grass) with buffalo dung in farm’s around community existing digester becomes a valid approach to enhance biogas production Also, the addition of grass can help raise C:N of the feedstock to be suitable for metabolic activities in anaerobic digestion system. The physical structure and chemical composition of lignocellulosic materials can be altered through various methods of pretreatment, breaking down the linkage between polysaccharides and lignin, thus making cellulose and hemicelluloses more accessible to hydrolytic enzymes (Wannapokin et al. 2018). Therefore, pretreatments could accelerate the hydrolysis process and improve the methane content in the biogas.

Strevett et al. (1995) stated that water vapor in biogas is problematic for compressibility and should be removed prior to storage. And biogas typically contains a high percentage of carbon dioxide (CO_2_), which decreases its caloric value. Finally, hydrogen sulfide (H_2_S), which is also present in biogas, is toxic and exhibits corrosive effects on process equipment if not removed prior to compression and storage. Physicochemical methods such as physical adsorption, physical absorption or chemical absorption are commonly used to treat biogas. However, these biogas purification methods require costly investment and maintenance which are not suitable for industrial scale and reduce the profit. Therefore, biological purification that takes advantages of photosynthesis process of plant such as microalgae to eliminate CO_2_ from biogas can be applied to reduce the capital and operations cost as enhance the biogas quality (Ramaraj et al. 2016a, b ,c). Therefore, this study main aim is to assess different pre-treatment and fermentation techniques through experimentation and evaluate each process and improvement of biogas yield. Finally, biogas production from buffalo grass (*Brachiaria mutica*) co-digestion with buffalo dung) through anaerobic enhanced methane content achieved by microalgae pass biological purification. Additionally, this study aimed to use non-food plant source as a feedstock for biogas production, a renewable energy fuel.

## Materials and methods

### Collection and preparation of substrates

**Fig. 1:**
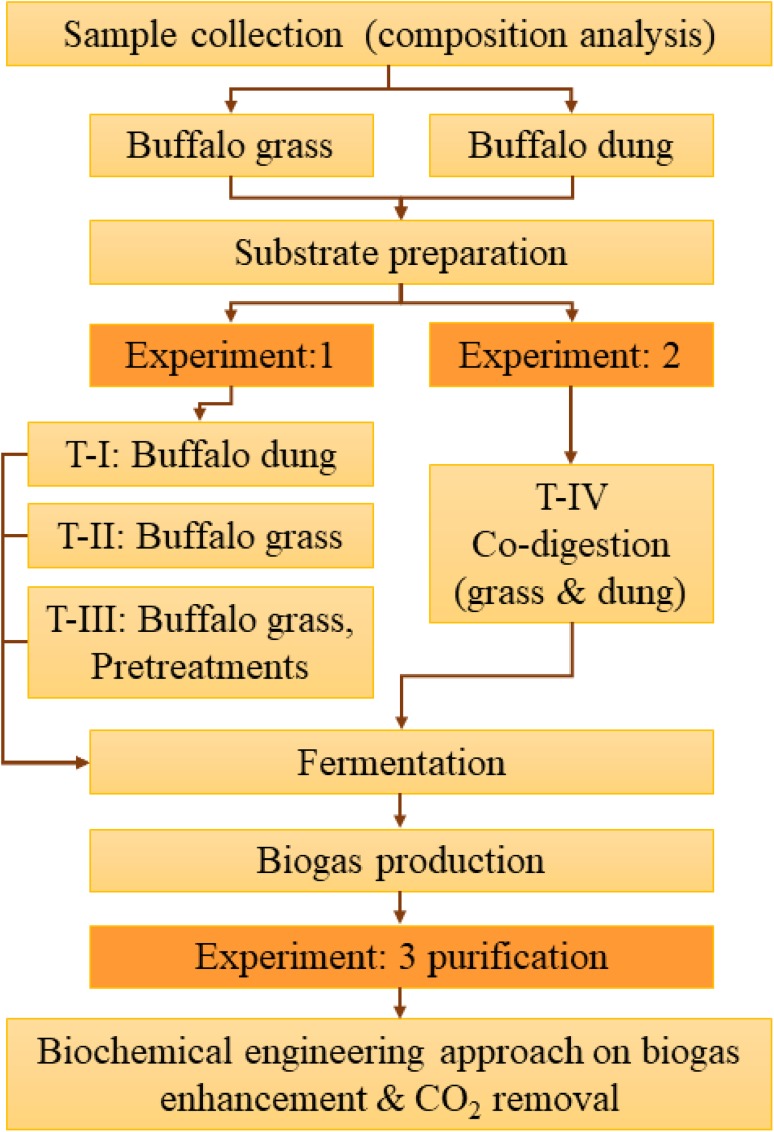
The flowchart of study methodology

The study methodology is illustrated in Fig. 1. This experimental study was carried out at an Energy Research Center (ERC), Maejo University, Chiang Mai, Thailand (18°53′35″N; 99°01′10″E); additionally, the buffalo grass and buffalo dung were collected near to the experimental zone. The grass sample was crushed by a machine into small particles. Stored grass was pulverized into small particles (1.0 mm) before use. The inoculum was utilized from the Maejo pig farms located at the University campus. For biogas purification, the microalgae were obtained from ERC and the culturing details were described by Ramaraj et al. (2016a, b ,c).

### Experimental setup

**Fig. 2:**
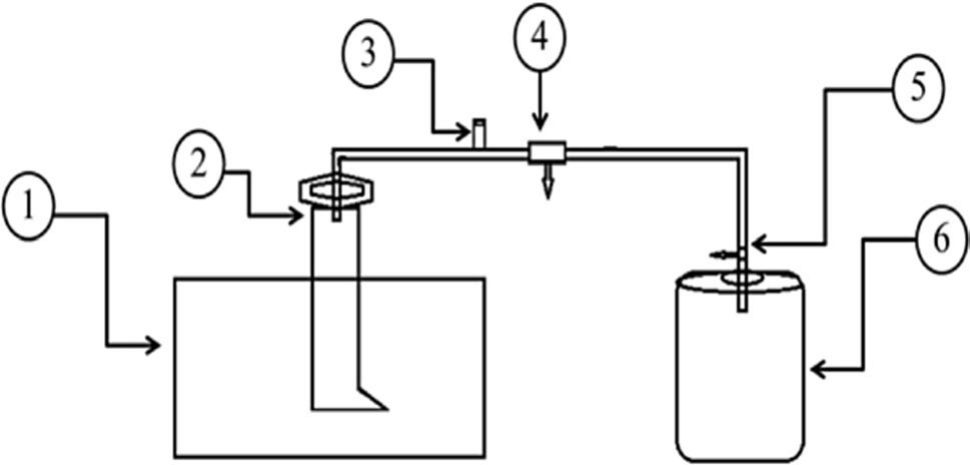
The digester (1) water bath, (2) gas holder, (3) gas release valve, (4) gas line connecter, (5) gas line tube and (6) fermenter

The buffalo grass was pretreated with boiling water at 100 °C with different reaction time ranging from 0.5 to 2 h. The experiments were carried out in batch type laboratory scale reactors and were categorized based on the different treatments applied: T-I (no treatment, buffalo dung), T-II (no TREATMENT, buffalo grass), T-III-A (buffalo grass, boiled 100 °C 0.5 h), T-III-B (buffalo grass boiled 100 °C 1 h), T-III-C (buffalo grass, boiled for 1.5 h at 100 °C), T-III-D (buffalo grass, boiled for 2 h at 100 °C) and T-IV (co-digestion of buffalo dung and buffalo grass, boiled for 2 h at 100 °C,). Experiment T-IV was operated with 1:1 ratio of grass and dung. Each reactor was made from a 7 L plastic container placed in a water bath. All reactors with 5 L working volume were run simultaneously for 35 days. The schematic configuration of the anaerobic biogas reactor system is given in Fig. 2. The accumulated biogas was stored carefully until the sufficient volume for purification experiments was reached.

### Analytical methods

Parameters such as total solid (TS), volatile solids (VS), fixed solids (FS), chemical oxygen demand (COD), ash and moisture contents were measured according to the standard methods (APHA 2005). The compositions of sample (cellulose, hemicellulose, and lignin) were determined by Van Soest method (Van Soest et al. 1991). Metrohm 774 pH meter was used in all pH measurements. The pH was adjusted ranging from 7.40 to 7.70 for all experiments. Direct titration method for the determination of total volatile fatty acids and alkalinity was used (Ennouri et al. 2016). Samples were titrated with 0.1 N HCl (pH = 3), boiled over 3 min to remove CO_2_, then back-titrated using 0.1 N NaOH until the pH reached 6.5. The biogas volume produced from the batch digester was determined using a water displacement unit. The pH of the substrate and digestate was determined using pH meter. The concentration of methane (CH_4_) and other gases including carbon dioxide (CO_2_), hydrogen sulfide (H_2_S), and oxygen (O_2_) in biogas produced were all determined by a portable gas analyzer (BIO5000, UK). The volume of biogas produced was measured at daily basis and biogas compositional analysis was performed every 3 days. The samples were analyzed for organic carbon, nitrogen (alkaline KMnO_4_ method), 0.5 M NaHCO_3_ (pH 8.5) extractable P and 1 (N) NH4OAc—extractable K and other trace elements (Page et al. 1982). In addition, emission, atomic absorption, volumetric, colorimetric, and photometric methods were used to determine physicochemical digestate properties and measurements adopted from Kinyua et al. (2016).

Calorific values were estimated according to Li et al. (2014).The higher calorific values (HCV) and lower calorific values (LCV) of pure methane were 39.82 and 35.87 MJ/m^3^, respectively. HCV and LCV of produced biogas were determined according to the following formula:

1 $${\text{HCV}}_{\text{biogas}} = 0.3989 \times {\text{MC}} = 0.0213({R^2 = 1})$$

2 $${\text{LCV}}_{\text{biogas}} = 0.3593 \times {\text{MC}} = 0.0192({R^2 = 1})$$

where MC is the methane content in biogas (%).

### Characterization of pretreated and untreated biomass

The biomass was characterized using scanning electron microscope, in order to observe the changes on the structure before and after applying pretreatment, characterization of biomass was done analysis using scanning electron microscopy analysis (JSM–5410LV, USA). The observation was performed at a total magnification of 100 ×.

### Biogas through biological purification

**Fig. 3:**
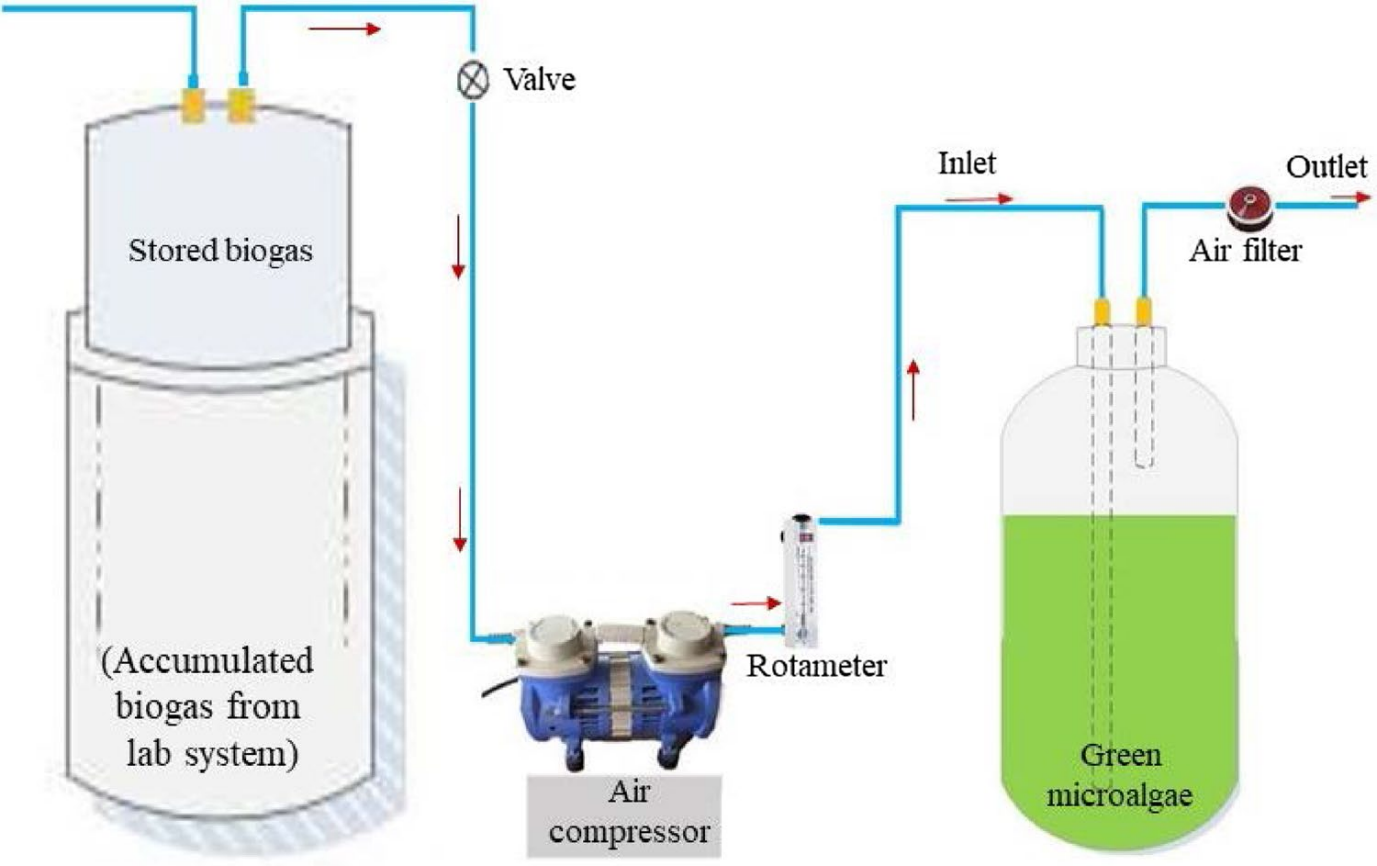
Biogas enhancement through biological purification system

Biogas enhancement was performed through photoautotrophic microalgae (*Chlorella vulgaris*). The experiment was continued for 8 h. Two types of biogas flow rate (0.9 and 1.8 lpm) in the algae growth unit were applied. The biological biogas purification process is described in Fig. 3.

### Statistical analysis

The values reported in the present study were the mean of three replicates. Statistical analyses of data were performed using the program SPSS 20.0 (SPSS Inc., Chicago, IL, USA). A significant difference was considered at the level of *p* < 0.05.

## Results and discussion

### Substrate characteristics

Feedstock characteristic is an important factor influencing digester’s performance and stability. Buffalo grass (*Brachiaria mutica*) commonly known as Para grass is a member of the Poaceae family which is found as aquatic weeds throughout northern part of Thailand. Buffalo grass is estimated to contain about 40–44% of cellulose, about 18–22% hemicellulose and 18–21% of lignin. The initial pH, ash and moisture were 8.26, 2.79 and 77.3%, respectively. TS, VS, COD, alkalinity, volatile fatty acid were 349,813 mg/l, 128,275 mg/l, 62,333 mg/l, 2733 mg/l–CaCO_3_, 4013 mg/l, respectively. The characteristics of buffalo dung TS, VS, COD, alkalinity, volatile fatty acid, pH, ash and moisture were 246,397 mg/l, 195,253 mg/l, 30,333 mg/l, 2400 mg/l–CaCO_3_, 1260 mg/l, 8.02, 2.9 and 83.0%, respectively.

### Imaging with scanning electron microscopy (SEM)

**Fig. 4:**
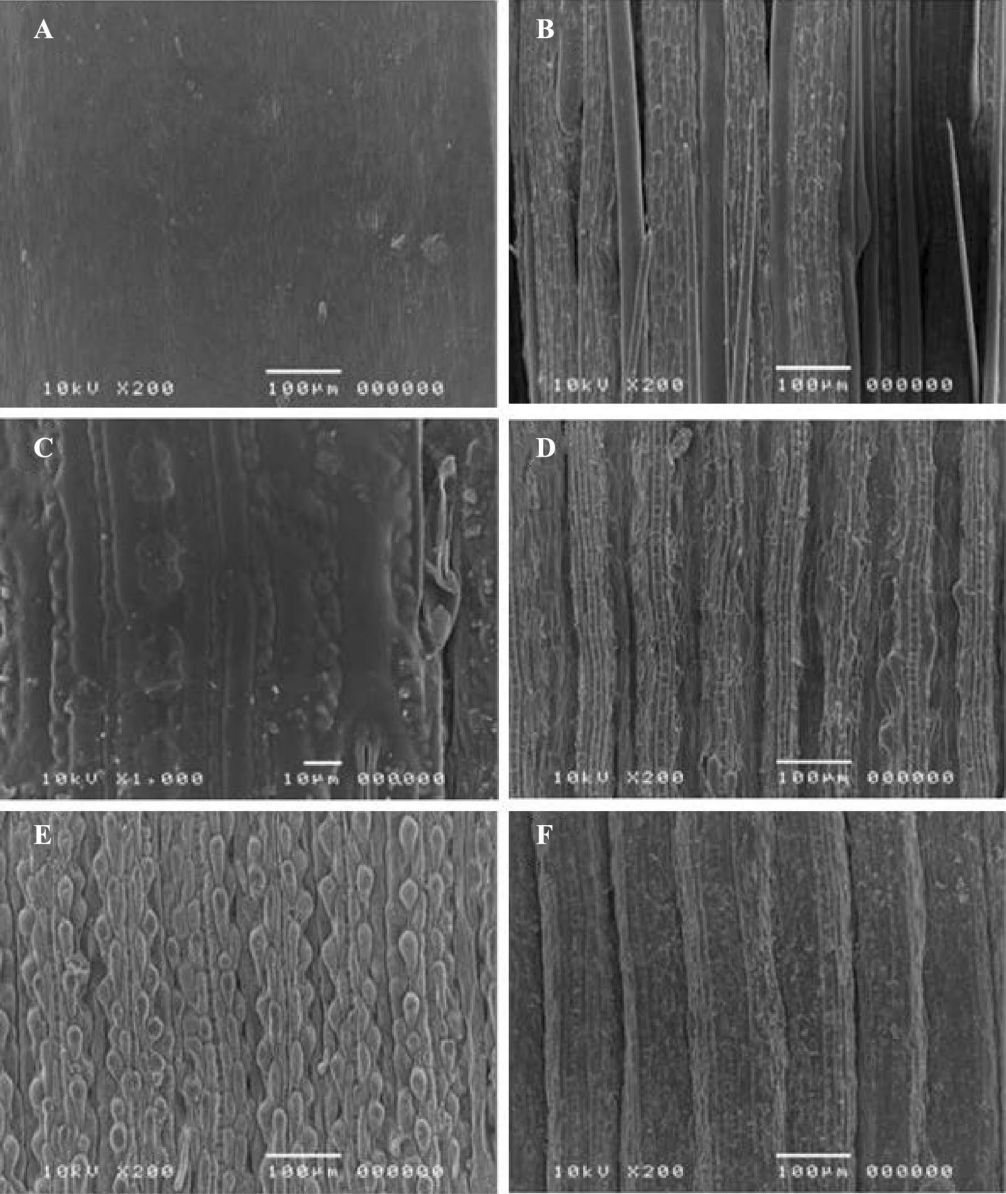
Scanning electron micrographs of morphological characteristics of non-pretreated and pretreated of buffalo grass samples: **a** stem (not pretreated), **b** stem pretreated by boiling, **c** upper leaf epidermis (not pretreated), **d** pretreated upper leaf epidermis, **e** lower leaf epidermis (not pretreated) and **f** pretreated lower leaf epidermis

Morphological changes in the treated and untreated Buffalo grass during the hydrothermal pretreatments were observed using scanning electron microscope. SEM analyses was carried out to assess changes in morphology of the native and pretreated samples boiled at 100 °C with 2 h retention time. Figure 4a shows the SEM micrograph of native buffalo grass stem, the surface of which shows to have a regular and compact structure. Morphological changes induced by boiling are first noticeable after a pretreatment on buffalo grass stem, as shown in Fig. 4b.

A slight defibrillation was observed (shown in Fig. 4c, d); the separation of individual fibers, enlargement of the reactive area and more pronounced structural changes in the biomass were seen due to a possible solubilization of the hemicellulose. As hemicellulose operates as a cementing material, its solubilization causes a significant defibrillation effect on the biomass. In addition, a reduction in fiber length and the formation of entangled clusters can be seen in Fig. 4e, f; the fiber structure was almost entirely disintegrated due to the higher solubilization of hemicellulose and lignin re-localization. It was found that the fibers were greatly affected by boiling with 2 h retention time. In addition, the swelling of fibers is also observed in boiling pretreated biomass. This result was also supported by the structural changes observed from the SEM images of the stem, upper and lower leaf epidermis of the buffalo grass samples.

### Pretreatment and biogas production

Hydrothermal pretreatment in lignocellulosic feedstock involves the usage of water only and has been widely accepted as a green technology without potential chemical consumption and potential pollution (Saha et al. 2013). Typically, it can remove most of hemicellulose and part of lignin in biomass by degrading them into soluble fractions and loosening the recalcitrant structure as well (Li et al. 2017). Therefore, hydrothermal pretreatment has been widely applied for facilitating biofuels production (Cybulska et al. 2014). They have long been used for enhancing particulate organic matter disintegration at temperatures from 50 to 270 °C. This study was applied with boiling pretreatment. Batch anaerobic fermentation was conducted to study the biogas potential of boiling preferment with mono and digestion of buffalo grass with buffalo dung. These experimental results are presented in Table 1. With 100 °C boiling water, the buffalo grass produces higher biogas yield and methane content by retention time (i.e., T-III-A < T-III-B < T-III-C < T-III-D = 58.13% CH_4_ < 62.17% CH_4_ < 63.78 CH_4_% < 66.10 CH_4_%. Furthermore, accumulated biogas yield was increased along with retention time. As a study result, the main functions of hydrothermal pretreatment on converting the insoluble components into soluble fractions, breaking physical structure, and homogenizing feedstock sizes may improve anaerobic digestion.

**Table 1:** The effect of pretreatment and biogas yield

Items		Parameters				
		CH_4_ (%)	CO_2_ (%)	O_2_ (%)	H_2_S (ppm)	Accumulated biogas yield (ml)
No treatment, dung	T-I	52.27	42.8	0.1	454 (0.0454%)	8982
No treatment, grass	T-II	50.34	44.5	0.1	403 (0.0403%)	7184
Boiled 100 °C 0.5 h (grass)	T-III-A	58.13	39	0.1	384 (0.0384%)	9522
Boiled 100 °C 1 h (grass)	T-III-B	62.17	37	0.1	331 (0.0331%)	10,975
Boiled 100 °C 1.5 h (grass)	T-III-C	63.78	35.4	0.1	234 (0.0234%)	11,047
Boiled 100 °C 2 h (grass)	T-III-D	66.10	33	0	217 (0.0217%)	13,185
Co-digestion of grass (boiled 100 °C 2 h) and dung	T-IV	71.00	28	0	132 (0.0132%)	15,521

**Fig. 5:**
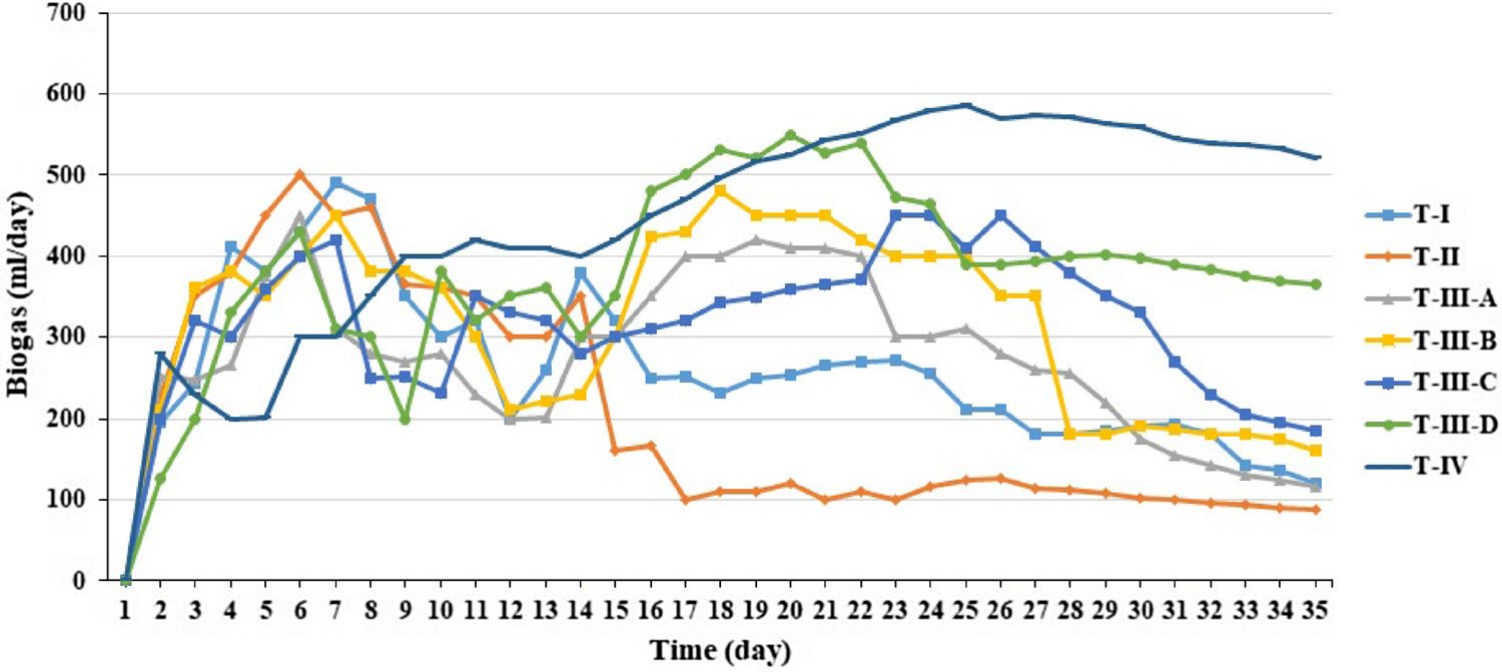
Daily biogas production

**Fig. 6:**
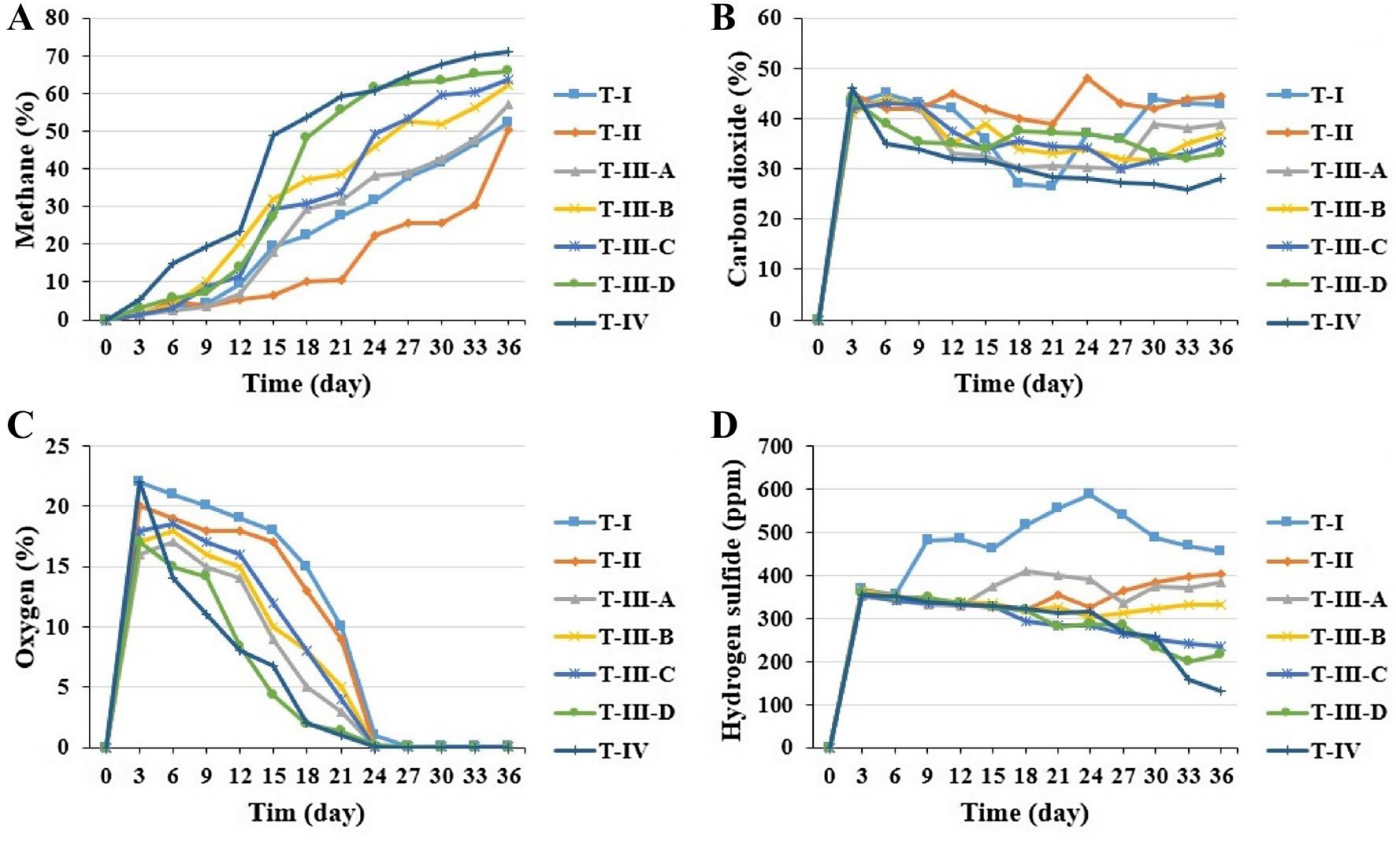
Biogas composition: **a** methane, **b** carbon dioxide, **c** oxygen and **d** hydrogen sulfide

The methane production rate reflects the biodegradability and amount of degradable matter. The daily biogas and gas composition including methane, carbon dioxide, hydrogen sulfide and oxygen production characteristics is shown in Figs. 5 and 6. Codigestion is defined as the digestion of mixtures of at least two waste materials for improving AD efficiency. Many successful codigestions of substrates have increased methane potential substantially compared to the mono digestion of the substrates (González–Fernández et al. 2011; Teghammar et al. 2013). These study results clearly demonstrated and agreed with González–Fernández et al. (2011) and Teghammar et al. (2013). Co-digestion of buffalo grass and buffalo dung produced higher accumulated biogas (15,521 ml) and rich methane content (71%) compared to mono digestion.

**Fig. 7:**
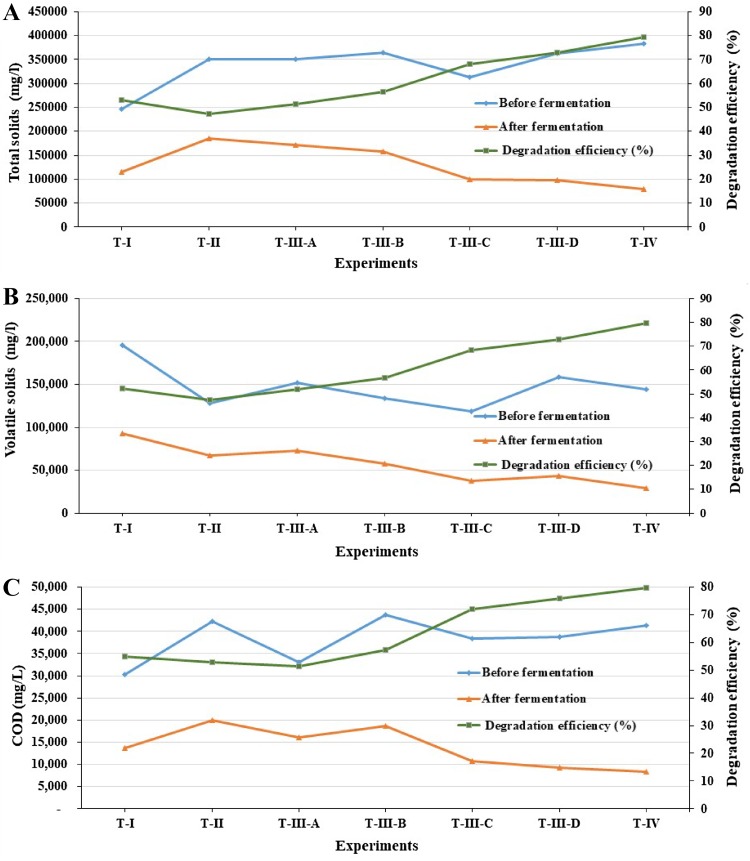
Total solids, volatile solids and chemical oxidation demand of before and after fermentation

The total solids, volatile solids, chemical oxidation demand, alkalinity, volatile fatty acid and pH performance on before and after fermentation process was presented in Table 2 and Fig. 7. VFA formed during the acid phase of the anaerobic digestion tends to reduce the system pH, making the methanogenic bacteria, which are sensitive to low pH values, reduce their activity (Zhang et al. 2008). Thus, a balance between the production and consumption of acid during the refuse biodigestion is essential for the stability of the anaerobic process. The pH is one of the key factors in AD and the growth of methanogens can be significantly influenced by the pH level. VFA can maintain an efficient AD performance by influencing pH levels and alkalinity. The determination of volatile solids is a good parameter to follow the biodegradable organic matter degradation and its analysis is commonly applied to the biological stability measurement in sludge from liquid effluents (Metcalf and Eddy 1991). The anaerobic stabilization process starts when the volatile suspended solids of the system are hydrolyzed, resulting in soluble COD. The soluble COD represents the soluble organic matter of the system, which in turn is substrate for the methanogenesis, being converted into CH_4_ and CO_2_ (Zhang et al. 2008). Carbon is among the main nutrients for the microorganisms, as it is a source of energy for the microbial population; nitrogen is crucial for the microbial population growth (Igoni et al. 2008). Despite the volatile solid values being still relatively high at the end of the process, the final carbon values reveal that the biogas production develops to the end in the biodigesters; the TS, VS, and COD degradation efficiency were 79.48, 79.72 and 79.80%, respectively, which were consumed within the 35 days of the biodigestion.

**Table 2:** Alkalinity, volatile fatty acid and pH performance on before and after fermentation

Treatments	Alkalinity (mg/l–CaCO_3_)		Volatile fatty acid (mg/l)		pH	
	Before fermentation	After fermentation	Before fermentation	After fermentation	Before fermentation	After fermentation
T-I	2400	3833	3960	3844	7.55	7.06
T-II	2733	3133	4013	3820	7.55	6.53
T-III-A	2533	3800	4166	3912	7.55	6.55
T-III-B	2767	3233	4058	3949	7.55	6.54
T-III-C	2935	3324	4195	42,477	7.55	6.51
T-III-D	2787	3143	4004	3990	7.55	6.54
T-IV	2948	3072	4123	4246	7.55	6.52

### Biogas enhancement through biological process

There are a number of purification methods that have been applied in some countries, namely absorption of liquids into the physics/chemical; adsorption on the surface of a solid adsorbent, membranes separation, cryogenic separation, and chemical change. However, these technologies showed that there is a high cost to purify biomethane, which is three times higher than that of the biogas production cost. An alternative technique to upgrade biogas is to use photosynthetic CO_2_ uptake by microalgae. Microalgae have high carbon fixation ability and rapid growth rate, and can be adapted to various environmental conditions (Ramaraj et al. 2016a, b ,c). When microalgae are utilized for biogas upgrading, the photosynthesis can efficiently convert CO_2_ in raw biogas into its biomass (Tang et al. 2011). This allows the valorization of biogas CO_2_ in the form of a valuable microalgae biomass, which can be used as feedstock to produce biofuels or even high value-added by-product. In this study, biogas purification and methane enhancement through biological process are presented in Table 3.

**Table 3:** biogas purification and methane enhancement through biological process

Parameters	Performance	Biogas composition (%)					
Biogas composition	Biogas flow rate	CH_4_ (%)	CO_2_ (%)	O_2_ (%)	H_2_S (%)	Other trace gases (%)	References
Before purification	**–**	68.8	29.7	0	0.077	**–**	Dussadee et al. (2014)
After purification	**–**	89.35	10.05	0.02	0.001	**–**	
Before purification	**–**	64.67	31.5	0	0.058	**–**	Ramaraj et al. (2016a, b ,c)
After purification	**–**	82.05	17.08	1.11	0.001	**–**	
Before purification	–	71	28	0	0.013	0.99	This study
After purification	0.9 lpm	91	8.56	1.49	0	0.11	
	1.8 lpm	83	15	1.31	0	0.65	

Gupta et al. 2014 revealed that H_2_S might lead to the inhibitory effect on photosynthesis in the bioreactor system. In this is case, the study biogas does not have H_2_S. Therefore, the inhibitory impact of H_2_S on photosynthesis process that is relevant to biological purification using microalgae was ignored. Basically, SO_3_^2−^ is known to inhibit photosynthetic CO_2_ fixation in plants due to SO_3_^2−^ outcompeting CO_2_ in rubisco and inhibit mitochondrial ATP production and this study system does not meet this situation due to the lack of H_2_S. Also, H_2_S concentrations present in raw biogas up to 3000 ppmv did not exert notable inhibitory effects on microalgae growth (Yan et al. 2016).

Since the metabolism and photosynthesis of microalgae depend on microalgae growth, the law of nutrient and CO_2_ removal efficiency changed as well as the variation tendency of microalgal growth.  Furthermore, this study results revealed that flow rate as a vital factor for biogas purification. Different flowrates (0.9–1.8lpm) were achieved methane content of 83%–91%, and other biogas components were demonstrated in Table 3. In addition, biogas flow rate (1.8 lpm) exposed the better performance compared to the previous studies (Dussadee et al. 2014; Ramaraj et al. 2016a, b ,c). Zhu (2015) was confirmed that CO_2_ in biogas can be used as an important carbon source for microalgae cells growth. Also it is not difficult to conclude that N and P are more insufficient than carbon sources during the growth of microalgae according to the nutrient removal efficiency results. For the same reason, the CO_2_ in the biogas was consumed during the photosynthesis of microalgae, so the biogas purification capacity was also improved.

### Enhanced biogas calorific value and digestate fertilizer

Enhanced biogas (from co-digestion of buffalo grass and buffalo dung) HCV was 36.30 MJ/m^3^ and LCV was 32.70 MJ/m^3^. It was much higher than biogas production from traditional AD (LCV of 18.0–23.4 MJ/m^3^ and HCV of 20.0–25.9 MJ/m^3^) (Li et al. 2014); accordingly, these study results verified that high-calorific biogas was obtained in this study system after methane was enriched through biological biogas purification. Finally, the digestate from codigestion of buffalo grass and buffalo dung was analyzed. The study digestate and the literature data are presented in Table 4. Digestate can be defined as liquid from anaerobic decomposition of animal and plant waste. It contains considerable amounts of mineral elements including nitrogen, phosphorus, potassium and others. In terms of rapidity of action, it resembles mineral fertilizers since N, P and K elements are easily available for plants. Govasmark et al. (2011) and Heviánková et al. (2013) proved the possibility of occurrence of pathogenic bacteria and heavy metals in digestate. This is why it is important that digestate is safe for use as a fertilizer and also highlighted the use of digestate as a fertilizer in place of mineral fertilizers (Vázquez–Rowe et al. 2015). Na concentration is an important factor to assess the suitability of effluent irrigation. Phosphorus is essential for microorganism growth. Based on the results obtained in this research, an alternative to mitigate those problems is using biogas digestate, which could supply the chemical fertilizer demands.

**Table 4:** Chemical compositions of digestate from the different anaerobic digesters

Raw materials	TOC, g L^−1^	N	P	K	S	Ca	Mg	Fe	Mn	Zn	Cu	References
		g kg^−1^ in the form of dry matter										
Poultry manure	452	67	24	24	5.3	92	6	1.8	0.66	0.58	0.11	Kirchmann and Witter (1992)
Biodegradable household waste	ND	152	16	78	7	50	10	ND	< 0.001	0.08	0.01	Haraldsen et al. (2011)
Pig manure + sludge from wastewater treatment plant + biodiesel wastewater	247	200	6	52	ND	26	10	1	0.16	1.16	0.21	Alburquerque et al. (2012)
Maize silage	ND	41	34.8	5.9	ND	3.7	36.2	ND	ND	0.08	0.08	Pokój et al. (2015)
Co-digestion of buffalo grass + buffalo dung	389.17	77.53	13.39	37.86	5.73	35.76	12.11	2.14	0.36	0.75	0.19	This study

*ND* not determined

## Conclusions

In the present study, buffalo grass has been established as an efficient cosubstrate for buffalo dung to enhanced biogas production. While buffalo grass is a menacing aquatic biomass, it could also serve as an effective aquatic energy crop with controlled growth and proper maintenance in constructed wetlands and thus reduce the dependency of terrestrial energy crops for bioenergy generation in the near future. More specifically, the methane concentration from the co-digestion mixture was found to be the key parameters for an improved biomethanation process. The microalga biological purification of biogas enrichment was achieved successfully. Furthermore, the digestate from biogas fermenter was confirmed to be an efficient alternative fertilizer with high nutrients and environmentally-friendly comparing to chemical fertilizer.
